# Image deformation as a cue to material category judgment

**DOI:** 10.1038/srep44274

**Published:** 2017-03-09

**Authors:** Takahiro Kawabe, Rok Kogovšek

**Affiliations:** 1 NTT Communication Science Laboratories, Nippon Telegraph and Telephone Corporation, Japan; 2 Faculty of Computer and Information Science, University of Ljubljana, Slovenia

## Abstract

Human observers easily recognize complex natural phenomena, such as flowing water, which often generate highly chaotic dynamic arrays of light on the retina. It has not been clarified how the visual system discerns the source of a fluid flow. Here we show that the magnitude of image deformation caused by light refraction is a critical factor for the visual system to determine the perceptual category of fluid flows. Employing a physics engine, we created computer-rendered scenes of water and hot air flows. For each flow, we manipulated the rendering parameters (distortion factors and the index of refraction) that strongly influence the magnitude of image deformation. The observers rated how strongly they felt impressions of water and hot air in the video clips of the flows. The ratings showed that the water and hot air impressions were positively and negatively related to the magnitude of image deformation. Based on the results, we discuss how the visual system heuristically utilizes image deformation to discern non-rigid materials such as water and hot air flows.

Vision scientists have recently attempted to unveil the perceptual and cognitive mechanisms underlying the recognition of materials^1^. Most of them have focused on the perception of material surface properties. In particular, previous studies have shown that human observers can decode surface reflectance properties such as specularity^2^,^3^,^4^,^5^,^6^ and subsurface scattering^7^,^8^ from complex retinal images.

In addition to surface reflectance, surface refraction is an important phenomenon determining perceptual appearance of materials. When light penetrates a transparent material with an index of refraction (IOR) of more (or less) than 1, the path of the light is bent at the material’s surface, where the direction of the bending depends on the IOR. The bent light path often causes image deformation of the scene behind a curved material, wherein the larger (smaller) IOR of the material creates a larger (smaller) magnitude of image deformation on the retina.

It has been reported that image deformation due to refraction is a vital cue for the visual system to judge several aspects of material properties. For example, the magnitude of image deformation serves as a cue to judge the thickness of rigid materials such as glass [ref. 9, but also see ref. 10]. Moreover, the human visual system can utilize the dynamic pattern of image deformation due to refraction as a cue to a material’s elasticity^11^.

Importantly, dynamic image deformation gives observers the impression of a transparent water-like layer. A previous study^12^ reported that human observers can recognize a transparent water flow from dynamic image deformation alone; no other cues, such as specular reflection or luminance reduction due to absorption, were necessary for the transparent water recognition. It also showed that the critical component in dynamic image deformation is the spatiotemporal frequency of deformation and suggested that the specific band of the spatiotemporal deformation frequency is critical for perceiving the deformation as arising from the intervention of a transparent water layer.

Besides a water flow, a hot air flow also optically deforms the image behind it. A temperature change physically causes the spatiotemporal variation of the density of air, which eventually alters the IOR. Because the density change spatiotemporally occurs on the basis of the Rayleigh–Taylor instability^13^, the image deformation caused by refraction can be dynamic. In our everyday experience, we easily notice the existence of hot air just by seeing the dynamic image deformation. On the other hand, how the visual system recognizes hot air and how the system differentiates between hot air and water flows on the basis of dynamic image deformation are still open questions. Clarifying how the visual system categorizes transparent non-rigid material will be an important step to understanding how the mind generates rich and detailed visual representations of the very complex real world from a handful of visual inputs.

In this work, we examined what the critical image information is for human observers to discern the source of a transparent material flow. We used computer graphics with a physics engine to create video clips of simulated hot air flows and water flows. We noticed that the former generated a smaller magnitude of image deformation than the latter. Thus, we parametrically manipulated the magnitude of image deformation by changing rendering parameters. We then found that the hot air and water impressions were negatively and positively related to the magnitude of image deformation under the condition where other aspects of the flows, such as flow direction and turbulence, were intact. On the basis of the results, we propose a visual mechanism underlying the recognition of fluid materials from image deformation.

## Results and Discussion

**Figure 1:**
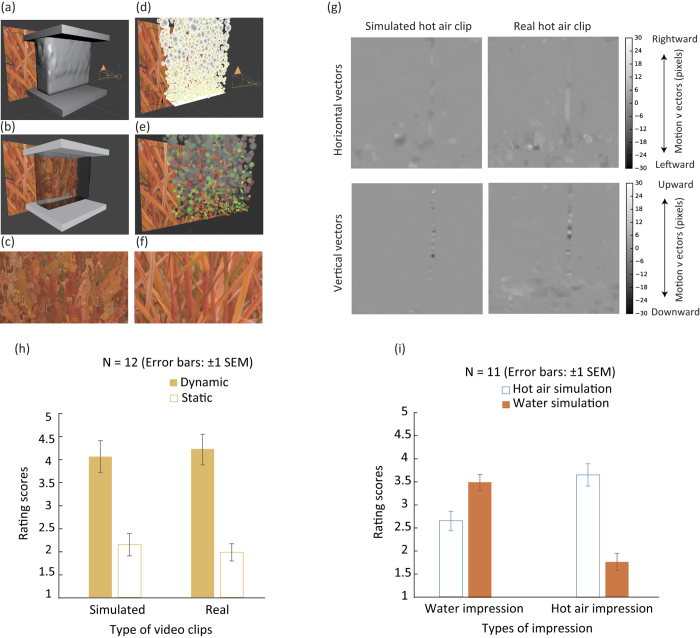
Simulation geometry, rendered scene, motion vector analysis, and experimental results. (**a**) The geometry of water simulation seen from an oblique angle. (**b**) A rendered scene of (**a**), which was viewed from an oblique angle. (**c**) A rendered scene of water flow. (**d**) The geometry of hot air simulation seen from an oblique angle. (**e**) A rendered scene of (**d**), which was viewed from an oblique angle. (**f**) A rendered scene of hot air. (**g**) Motion vector comparison between clips of simulated and real hot air for horizontal (upper row) and vertical (lower row) vectors, which were calculated between two video frames. (**h**) Experiment 1 results. (**i**) Experiment 2 results.

In Experiment 1, we first tried to determine whether our simulation of hot air (Fig. 1a–f) could produce a reasonable level of the desired impression. The observers were asked to rate their hot air impression after they had watched the video clips of simulated and real hot air flows. As shown in Fig. 1g, the range of motion vectors was similar between the simulated hot air and real hot air. Two styles of video clip presentation were tested. One was a dynamic style in which dynamic sequences of video frames were presented. The other was a static style in which a single static video frame was randomly extracted from a sequence of video frames and presented to the observer. The results (shown in Fig. 1h) showed that the hot air impression in the simulation could produce a comparable level of impression to the clips of real hot air. A two-way repeated measures ANOVA with clip types and presentation style as within-subject factors showed that there were no significant differences in rating scores between the simulation clips and real hot air ones [*F*(1, 11) = 0.000, *p* > 0.05, partial *η*^2^ = 0.000] (their average scores were just identical to each other by accident). The rating scores were strongly affected by the presentation style [*F*(1, 11) = 13.885, *p* < 0.005, partial *η*^2^ = 0.558], and this suggests that, consistent with the previous study^12^, dynamic image deformation is necessary for human observers to have the impression of transparent materials. Interaction between the type of video clips and the presentation style was not significant [*F*(1, 11) = 3.868, p > 0.05, partial *η*^2^ = 0.260]. The results indicate that our hot air simulation clips could give the observers the intended impression, whose strength was comparable to their impression of the clips of real hot air.

In Experiment 2, we wanted to check whether the observers could discern hot air from water in our simulation. To see the pure effect of image deformation on our task, our water flow simulation omitted specular reflection and caustics. In each trial, the observers were asked to view the clips of simulated water or hot air flows (Video 1) and successively rate their impressions of water and hot air. The results (Fig. 1i) showed that the water impression was stronger for water simulation clips [*t*(11) = 2.49, *p* < 0.04, *Cohen’s d* = 1.12] and that the hot air impression was stronger for hot air simulation clips [*t*(11) = 6.39, *p* < 0.0001, *Cohen’s d* = 1.63]. The results indicate that our simulation video clips can evoke the desired material impressions in an observer.

Then the question is: what is the critical image information that the visual system uses to perceptually differentiate between water and hot air? At a glance, the magnitude of image deformation appears quite larger in the water simulation than in the hot air one (please compare the magnitude of image deformation between Fig. 1c and f). This is because the IOR for water is larger than for hot air. It therefore seemed promising to focus on the magnitude of image deformation as a cue the visual system uses to discern the source of a transparent material’s flow.

To this end, we calculated the optical flow fields using the pyramidal iterative Lucas-Kanade method^14^. We spatially trimmed the central 512 × 512 pixel region in the simulation video clips and temporally extracted 30 sequential frames. We then calculated the optical flow fields with the sequence of images having the structure of 512 (pixels) × 512 (pixels) × 30 (frames). Here we focused on the standard deviation of motion vectors as an index of the magnitude of image deformation. Basically, local motion vectors of image deformation due to refraction are in oscillation. Therefore, the standard deviation of motion vectors was a better index for the magnitude of motion vectors in the optical flow fields than the mean value of motion vectors, which would likely result in approximately 0.

**Figure 2:**
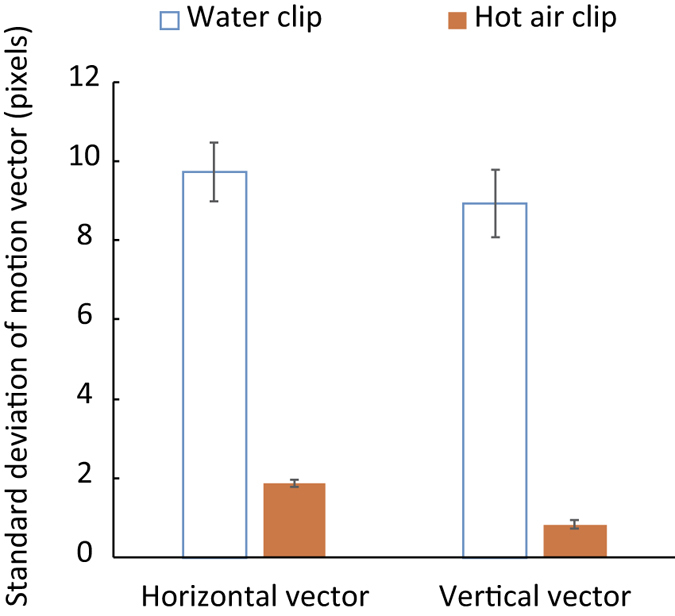
Standard deviation of motion vectors in the optical flow fields of simulated water and hot air. The simulation video clips with the background of a ‘Rocks’ scene (see Method section for details) were used for the calculation of optical flow fields. The standard deviation is separately shown for horizontal and vertical motion vectors. Error bars denote the standard deviation across 29 optical flow field patterns (which were obtained from each successive pair in 30 video frames).

The mean standard deviation of motion vectors in the calculated optical flow fields (Fig. 2) indicate that the video clips of simulated water produced a larger magnitude of image deformation than the video clips of simulated hot air. Thus, it is likely that the magnitude of image deformation is one of the critical factors determining the source of transparent flows, at least from an image processing perspective. On the other hand, it is unclear whether human observers could also utilize the magnitude of image deformation as a cue to differentiate between water and hot air.

**Figure 3:**
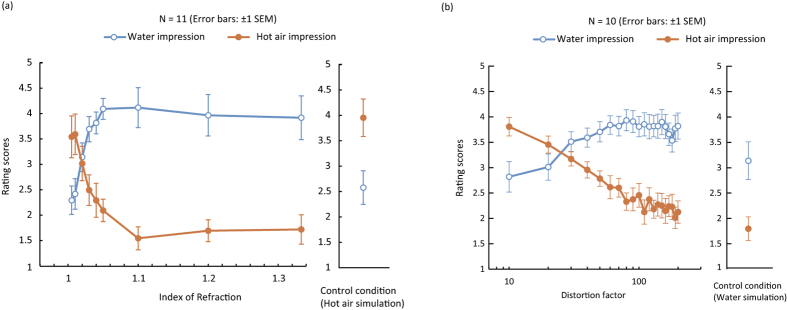
(**a**) Experiment 3 results. The left panel shows the relationship between the distortion factors and hot air/water impressions. The right panel shows the rating score in the control condition with the simulated hot air video clips. (**b**) Experiment 4 results. The left panel shows the relationship between the distortion factors and hot air/water impressions. The right panel shows the rating score in the control condition with the simulated water video clips.

Experiments 3 and 4 tested whether the magnitude of image deformation could influence the water and hot air impressions. In Experiment 3, we systematically manipulated the IOR in the water simulation and found that IOR strongly affected the water and hot air impressions (Fig. 3(a), Video 2). In particular, a lower IOR increased the hot air impression and decreased the water impression. For the hot air impression, a one-way repeated measures ANOVA with IOR (and control condition) as a within-subject factor revealed a significant main effect [*F*(9, 81) = 4.768, *p* < 0.0001, partial *η*^2^ = 0.346]. A multiple comparison test (Ryan’s method) showed that the rating values for IORs of 1.005 and 1.01 were significantly different from the rating values for IORs of 1.03, 1.04, 1.05, 1.1, 1.2, and 1.333 and the control condition (p < 0.05). For the water impression, the same ANOVA analysis as for hot air impression revealed a significant main effect [*F*(9, 81) = 6.894, *p* < 0.0001, partial *η*^2^ = 0.43]. The multiple comparison test showed that the rating values for IORs of 1.005 and 1.01 were significantly different from the rating values for IORs of 1.04, 1.05, 1.1, 1.2, and 1.333 (p < 0.05). Moreover, the rating values for IORs of 1.02 were significantly different from the rating values for IORs of 1.1, 1.2, and 1.333 (p < 0.05).

In Experiment 4, we systematically manipulated distortion factors in the hot air simulation (Fig. 3(b), Video 3) and found that the higher distortion factors attenuated the impression of hot air, while they boosted the impression of water. For the hot air impression, a one-way repeated measures ANOVA with distortion factors (and the control condition) as a within-subject factor showed a significant main effect [F(20, 200) = 14.437, p < 0.0001, partial η^2^ = 0.590]. The multiple comparison test showed that the rating values for distortion factors of 10, 20, and 30 were significantly different from the rating values for distortion factors of more than 30, 50, and 80, respectively (p < 0.05). The rating values for distortion factors of 40 were significantly different from the rating values for distortion factors of 110 and more than 130 (p < 0.05). The rating values for distortion factors of 50 were significantly different from the rating values for distortion factors of 200 (p < 0.05). For the water impression, the same one-way repeated measures ANOVA revealed a significant main effect [*F*(20, 200) = 4.206, p < 0.0001, partial η^2^ = 0.30]. The multiple comparison test showed that the rating values for distortion factors of 20 were significantly different from the rating values for distortion factors of more than 40 (p < 0.05). Moreover, the rating values for distortion factors of 30 were significantly different from the rating values for distortion factors of more than 70 (p < 0.05), but not for distortion factors of 180 and 190 (p > 0.05).

**Figure 4:**
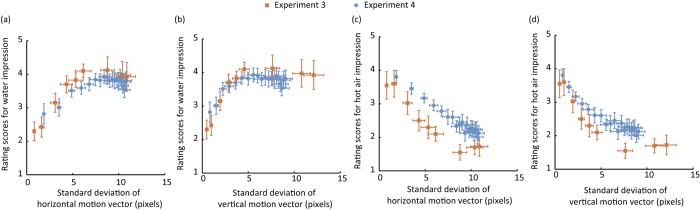
Plots of correlation between the standard deviation of motion vectors and rating scores: Rating scores for water impression as a function of the standard deviation of (**a**) horizontal and (**b**) vertical motion vectors. Rating scores for hot air impression as a function of the standard deviation of (**c**) horizontal and (**d**) vertical motion vectors. Each data point was obtained from each experimental condition tested in Experiments 3 and 4. Vertical error bars denote the standard error of mean across observers. Horizontal error bars denote the standard deviation of motion vectors across 29 patterns. The simulation video clips with the ‘Rocks’ scene background were used to calculate optical flow fields.

To directly assess relationships between the magnitude of image deformation and rating scores for the water/hot air impressions, we plotted the rating scores obtained in Experiments 3 and 4 as a function of the magnitude of image deformation (Fig. 4). The data clearly show that the rating scores were influenced by the standard deviation of the motion vectors as an index of the magnitude of image deformation. In particular, the larger and smaller image deformation magnitudes respectively contributed to the water and hot air impressions, indicating that the visual system utilizes the magnitude of image deformation as a cue for discerning the source of flows of transparent materials.

On the other hand, the relationship between the impression of a material and image deformation magnitude is not always linear. As the data show, the rating score did not change when the standard deviation of the motion vector exceeded the range of 5 to 8. The results indicate that certain levels of image deformation magnitudes are sufficient to trigger the impression of water.

In Experiment 3, as shown by the error bars, individual differences in the water impression increased when the IOR exceeded 1.1. Although the reason for the individual differences is not clear, we speculate that it might involve differences in the perceptual clarity of a transparent layer among the observers. Specifically, as the IOR increased, the spatial structure of background pattern is destroyed. Therefore, it might have been difficult for some observers to clearly separate a front liquid layer from a rear background pattern, which would explain the individual differences in the impression of transparent liquid.

We should mention that the level of rating scores was slightly different between Experiments 3 and 4. Specifically, in Fig. 4a and b, the rating scores for the water impression were slightly higher in Experiment 3, while in Fig. 4c and d, those for the hot air impression were slightly higher in Experiment 4. There are two possible explanations for the difference in the rating scores between the experiments. First, since different observers participated in the experiments, the criterion for rating might have also been different between the two groups of the observers. Second, in Experiment 3, the majority of the stimulus video clips were water simulation, while in Experiment 4, they were hot air simulation, and this might affect the criterion for the rating. If this is the case, there is a possibility that the observers can access image information other than the magnitude of image deformation to differentiate water and hot air flows. Water and hot air have different viscosities and so likely produce diverse image motion vector patterns in, for example, turbulence. Although the impression of the source of transparent flows is largely determined by the magnitude of image deformation, it is possible that other visual information also contributes to the recognition of transparent flows.

## General Discussion

In everyday life, we can tell water from hot air without an effort, but it has been unclear how the visual system accomplishes this trivial task. By combining graphical simulations of the transparent flow, image motion analyses, and psychological measurements, we found that the magnitude of image deformation is the strong determinant of the source of transparent flows in perception.

It is logically plausible to assume that in order to differentiate the source of a transparent flow on the basis of the magnitude of image deformation, the visual system needs to first recognize that an image deformation arises from an optical deformation due to light refraction at the surface of transparent materials, not from the physical deformation of scenes/objects themselves. We are likely to encounter the physical deformation of objects, such as a fabric waving in the wind, which often produces a large magnitude of image deformation Therefore, the visual system would not rely solely on the magnitude of image deformation to judge whether the image deformation arises from a water or a hot air flow. It is known that the visual system uses the spatial (and spatiotemporal) frequency of image deformation as a cue to interpret whether the image deformation comes from optical or physical deformation^12^,^15^. Specifically, human observers tend to report the existence of a transparent layer when an image deforms with relatively higher spatial frequency^15^. It is possible that our simulations of water and hot air flows satisfied the spatiotemporal frequency condition for the observers to see a transparent layer, and, in doing so, the observers might have been able to evaluate the magnitude of image deformation and differentiate the source of transparent flows.

As described in the introduction, a previous study has shown that the magnitude of image deformation contributes to the judgment of the thickness of a transparent rigid material^9^. Specifically, a transparent material causing larger image deformation is seen to be thicker than one causing smaller image deformation. Consistent with this suggestion, in our stimuli, the simulation of hot air flow, which involves a small image deformation, apparently produces a shallower depth impression than the simulation of water flow, which involves a large image deformation. Previous studies^9^,^10^ assessed the role of image deformation in the perception of the thickness of transparent materials has been assessed, but they did not ask observers to report what material they had perceived. In our study, the observers reported their impressions of material categories, but they were not asked to judge the thickness of each material. Thus, it is still unclear how the thickness of each material class is perceptually changed with the magnitude of image deformation.

There is a room to discuss whether we should have asked the observers to rate their impression of gas and liquid instead of hot air and water. Before the experiments, we discussed what we should ask the observers to do as a task. Consequently, we decided to ask for their water and hot air impressions because water and hot air are ubiquitous transparent materials in the environment. Thus, they are likely familiar to an observer (it seems it would be rather confusing for the observer to judge the impression of gas from image deformation because the most plentiful gas in our environment is air, which reflects light only slightly). On the other hand, it would possibly be a bit problematic if the observers perceived liquid and gas materials other than water and hot air, respectively. This is an open question for future studies to try to answer.

If observers could discriminate the materials by directly utilizing the IOR value, which is unique to each material, it would perhaps be pointless to ask observers to rate their impression of specific materials with varying IOR values. On the other hand, we do not always assume that a physical IOR value itself plays a strong role in the judgment of material categories; rather, we implicitly support the idea that human observers do not accurately infer the IOR of the materials from image deformation. Specifically, irrespective of the IOR, the observers might directly utilize image deformation magnitudes as the cue to material categorization. As described above, the change in the thickness of materials and/or in spatial frequency components of the background would easily alter the magnitude of image deformation and thus possibly alter the critical IOR values. This would in turn alter the impression from hot air to water, provided that the magnitude of the image deformation is a vital cue to material categorization by humans. Thus, we suggest that rather than the IOR, image deformation magnitudes are key factors to determining the material categorization. Based on that idea, we believe that our asking the observers to rate the impression of hot air and water is not problematic because we suppose that the observers would rely on the magnitude of image deformation in making their responses, not on the IOR value.

In our graphical simulation, we eliminated specular reflection and caustics from the water simulation in order to examine the pure role of image deformation in the differentiation between water and hot air. To differentiate them, it is highly likely that the visual system utilizes specular reflection and caustics, which do not occur in hot air. Here, it is an open question as to how the visual system integrates image deformation with specular reflection and caustics to see a coherent water flow. A previous study^16^ pointed out that the visual system is not sensitive to the spatiotemporal inconsistency between dynamic image deformation and specular flow. But it is still unclear how the inconsistency between them affects the categorization of the type of flow of transparent materials. In addition, in real-world scenarios, richer cues such as specular reflection are available to the visual system for categorizing a material. It would therefore be interesting to investigate how the visual system categorizes a transparent flow materials when it integrates several consistent (inconsistent) cues to its existence.

In addition to the magnitude of image deformation, other spatiotemporal image properties that covaried with image deformation might potentially determine the categorization between hot air and water. When comparing the appearance of static-style clips of stimuli between hot air and water, as used in Experiment 2, one might easily notice that the spatial structure of the background is distorted more strongly in the water clips than in the hot air ones. The difference in the magnitude of the spatial distortion might serve as critical information for discerning material categories on the basis of image deformation. It was also likely that the change in the image deformation magnitudes produced the change in local spatial frequency and orientation of the background image. Moreover, the variation in image deformation magnitudes caused the variation in image motion vectors which can be processed independently of shape information in the visual system. Thus, the effect of other image properties covarying with image deformation on the material categorization is another important issue to investigate in future studies.

That the liquid impression did not increase beyond the IOR of 1.1 in Experiment 3 might indicate that the spatial distortion itself could enhance the impression of liquid. In Experiment 3, we did not add a strong image blur to stimuli because we wanted to assess the effect of image deformation magnitudes on the impression of materials. On the other hand, the image deformation with large magnitude involves the spatial distortion of image information as described above. Thus, our observers might not have accepted our stimuli with weak blurriness but strong image deformation in static information as transparent hot air flows. In this respect, it is possible that such spatial (static) distortion cue plays a critical role in the categorization of materials from image deformation.

We also used only an upward flow of transparent materials. In general, image deformation in heated air goes upward because of the change in air density due to temperature increments. Considering the physical properties of image deformation due to hot air movement, we used only an upward flow of hot air to imitate the natural appearance of a hot air flow. For an experimental control reason, we also used an upward flow of water in order to match the flow direction between water and hot air. On the other hand, we preliminary observed that a downward hot air flow was sometimes seen as a shallow water flow, and this indicates that the flow direction of transparent materials is also likely a strong cue to the judgment of the category of transparent materials. Further examinations are warranted in future studies to explore how visual information other than image deformation serves the differentiation of transparent materials and how they interact with each other.

## Methods

### General method

#### Observers

All observers in this study were unaware of the specific purpose of the experiments. They reported having normal or corrected-to-normal visual acuity. Participants were recruited from outside of the laboratory and paid for their participation. Ethical approval for this study was obtained from the ethical committee at Nippon Telegraph and Telephone Corporation (NTT Communication Science Laboratories Ethical Committee). The experiments were conducted according to the principles laid down in the Helsinki Declaration. Written informed consent was obtained from all participants.

#### Apparatus

Stimuli were presented on a 21-inch CRT monitor (GDM-F500R, Sony) with a resolution of 1024 × 768 pixels and a refresh rate of 60 Hz. We linearized the luminance emitted from the monitor in a range from 0 to 132 cd/m^2^ using a photometer (OP200-E, Cambridge Research Systems). A computer (Dell Precision T1650 with Windows 7 32-bit OS) controlled stimulus presentation and data collection with Psychopy v1.83^17^,^18^.

#### Stimuli

We used Blender (https://www.blender.org/), a professional open-source 3D computer graphics software, as our physically based computer graphics engine. Blender simulations were built with a similar simulation system for hot air and water flows. The simulation geometry had a plane holding the chosen background image, hemisphere lightning, a camera directly facing the background plane, and a simulation source between the background and the camera (Fig. 1). The size of the simulation source was set to 5.5 × 2 m with heights different between simulations of materials due to different simulation systems. The distance between the background and source was 3 m, while the distance between the source and camera was 5 m. Together with the source depth of 2 m, the distance between the camera and background was 8 m.

##### Water simulation

Water simulation was based on Blender’s fluid simulation engine with the combination of transparent materials (Fig. 1a and b), with the contribution of specular and diffuse reflection components minimized as the previous study^12^ did. Therefore, we used zero specular and diffuse reflection components, and we also set shadow values to zero. The only material setting, the IOR, was set to 1.333, which is identical to the real physical property of water. A raytracing method was used to calculate light refraction at the surface of transparent water. To allow the simulation source to simulate a liquid without its being pressed against the domain wall, which would create an impression of water flowing over a solid transparent material such as glass, we created a domain of bigger dimensions than the source. The domain size was set to 6 × 6 × 6 m. The settings of the fluid domain and inflows and outflows were as follows. Inflow (water simulation source) and outflow elements, which initialized and removed fluid particles, were boxes with the height 0.5 m. The inflow was upward and its speed was 15 m/s. The speed value was chosen so as to visually match the speed of fluid flows between water and hot air. The fluid physics engine was set to a resolution of 150 with a 7-s simulation duration. Gravity was set to 9.81 m/s^2^ with 0.005 gravity compressibility on automatic grid levels. The kinematic viscosity was set at 1 × 10^−6^ m^2^/s. For the slip style, a partial slip at 20% was chosen with smoothing at one and two subdivisions. The fluid particles were set to 0. The spatial size of the whole simulation clip was 1280 × 800 pixels.

##### Hot air simulation

While Blender already incorporates water simulation as a feature with parameter settings, it does not have such a direct feature for hot air. To justify the apparent quality of simulated hot air, we modulated the appearance of our hot air simulation system until the appearance of the simulation video clips was similar to real hot air in video clips available on the internet. We used a simple particle system as the source, and we used post-rendering shader and randomization processes (in Blender’s Cycles engine), which caused realistic distortion of the image of the background plane. The base of the system source was a plane incorporating a particle system that set the gravity weight to zero and therefore emitted particles upward (Fig. 1d). The particle system used an initial Blender ICO sphere as a particle base but randomly resized it to a maximum of 30% with a 0.5 random parameter. Further size adjustment was done with a gradient that set the particles to grow to the set maximum size through their lifetime. The system was set to create 10,000 particles to fill the whole spatial area of the simulation video clip. The lifetime of the particles was set to 100 frames but was randomized with a 0.75 random setting. The emission locations were automatically jittered with a factor of 1. Since the shader generated a repetitive result across particles, we also set the random angular velocity of particles to 1. Considering the number of random factors, we used the same cached calculations across simulations in the same experiment or even across multiple experiments. Turbulence was added to the particle movement with strength set to 10 with size 0.5, Flow and Noise 0, and seed set to 49. For better movement control, a wind force field was incorporated. It had the shape of a plane with strength 10, flow 3, noise 0, and speed 66. Figure 1e shows the particle system combined with the shader process, which distorts a background image. Specifically, the intensity of red and green RGB channels of the particles determined the distortion of the pixels that were behind the particle, which is displayed as yellow contour disks in Fig. 1d. Connecting the effect of the coloring with the lifetime of the particle gave us a flow of randomly colored particles that start as a variation of green and red and their combination (yellow), while they slowly turn into a gray color that defined the area with no distortion. The intensity of red and green channels defined the size of the displacement on the horizontal and vertical axes, respectively. At this stage, a Gaussian blur was also applied to the distorted areas since genuine hot air tends to include some blurriness besides distortions. The strength of both the distortion and blurriness could be controlled by size parameters, which simply multiply the size of a given factor for the desired effect. Through comparison with the reference video clips of real hot air, we arrived at the conclusion that our system worked most naturally at around a distortion factor of 10 and blur factor of 0.5. The blurriness was more or less fixed around this factor, while the distortion factor heavily depended on the appearance of the clips of actual hot air. For the experiments, however, we fixed the values to 10 for distortion and 0.5 for blur. Although we introduced image blur to the hot air simulation to imitate physical hot air, the effect of the image blur on the hot-air impression is not so critical, as shown in Video 4. The spatial size of the whole simulation clip was 1280 × 800 pixels.

##### Background

Natural scene images were added as background to the simulated scenes. We chose ten images out of the rich collection in the McGill Calibrated Color Image Database^19^. The selected images can be seen in Supplementary Figure 1. Depending on experiments, we used all or part of the chosen ten images. In Experiment 1, we used a single static video frame of the video clips of actual hot air as background.

## Experiment 1. Comparison between clips of real and simulated hot air flows

### Observers

Twelve observers participated in this experiment.

### Stimuli

We used two types of stimulus video clips: Clips of real hot air and clips of simulated hot air. For the former, we downloaded a clip from the internet (https://www.youtube.com/watch?v=xcLtgde23Ec, all rights of this clip are reserved by FreeStockHD VideoClips), which respectively consisted of 661 video frames sampled at 30 frames per second (or fps). In an experimental session, the program temporally trimmed the clips into 30 frames (i.e., 1-s length). The program also spatially trimmed the clip into 600 × 600 pixels. The start position of the trimming was randomly determined for each trial. In the video clips of simulated hot air, a simulated hot air was included in the scene. We wanted to match the background image behind the real hot air with that in the simulated hot air. Hence, we extracted the first frame from the clip of real hot air and used it as the background in the clip of simulated hot air. Using the video frame as background was not so problematic because the deformation was not perceptually evident in a still image. The parameters in the simulation were adjusted so as to match the appearance between the two types of clips: blur factor was set to 0.5 and the distortion factor was set to 12.5. The total length of the video clips was 90 frames, and the experimental program trimmed the clip into 30 frames (i.e., 1-s length). The original spatial size of the simulation video clips was 1280 × 720 pixels. The program spatially trimmed the clip into 600 × 600 pixels. The start position of the trimming was again randomly determined on each trial. The trimming also served to eliminate black blobs that appeared along the edge of a rendered image from stimuli used in this and following experiments.

### Procedure

Each observer was tested individually in a lit room. They sat approximately 70 cm from the CRT display. A session started when the observer clicked a button in the GUI. In one second, the program started to present either a real or simulated hot air video clip. In the dynamic condition, a 1-s video clip was played. In the static condition, a single video frame extracted from the video clip was presented for 1 s. After the clip/image disappeared, a uniform black field was displayed for 0.25 s, followed by instructions for the rating task. The instructions used Japanese words describing hot air materials. The rating was done on a scale from 1 to 5, where 5 was a strong impression of hot air and 1 was no impression of hot air. Each observer performed 32 trials consisting of two types of video clips (real or simulated hot air), two types of presentation (dynamic or static), two types of background, and four repetitions. We collapsed the data across presentation, background, and repetitions in the analysis because these three factors were not critical for our purpose.

## Experiment 2. Quality validation of our simulation video clips

### Observers

Eleven observers participated in this experiment. Ten of them had participated in Experiment 1, but they were still naive as to the purpose of the experiment.

### Stimuli

We used video clips of simulated water and hot air as stimuli. As described in General Methods, both the water and hot air were given upward flows and rendered with one of ten natural images as background.

### Procedure

The procedure was identical to that in Experiment 1 except for the following. After the disappearance of stimuli, the word indicating water was presented, and the observers were asked to rate their impression of water. Each rating was done on a scale from 1 to 5, where 5 meant the impression exactly corresponded to the material indicated by the displayed word, 3 was an inexact impression, and 1 meant that the impression did not correspond at all to the material described by the word. After they had reported the water impression, the word indicating hot air was presented, and the observers rated their impression of hot air. Each observer received 80 trials consisting of two types of video clips, ten types of background, and four repetitions. We collapsed the data across backgrounds and repetitions in the analysis because these two factors were not critical for our purpose.

## Experiment 3. IOR manipulation in water simulation

### Observers

Ten observers participated in this experiment. None had participated in the previous experiments.

### Stimuli

We used the video clips of simulated water flow as stimuli. Here, we manipulated the index of refraction (IOR) in nine levels (1.005, 1.01, 1.02, 1.03, 1.04, 1.05, 1.1, 1.2, and 1.333). The IOR manipulation drastically affected the magnitude of image deformation due to refraction; the larger the IOR was, larger the image deformation became. As stimuli in a control condition, we also used the video clips of hot air flow. We used a ‘Rocks’ image (the leftmost image in the upper row in Supplementary Figure 1 as background in both the water and hot air simulations.

### Procedure

As in Experiment 2, we asked the observers to sequentially rate their impressions of both the water and hot air. Each observer received 40 trials consisting of 9 levels of IOR × 4 repetitions and an additional four repetitions of simulated hot air. We collapsed the data across repetitions in the analysis.

## Experiment 4. Distortion factor manipulation in hot air simulation

### Observers

Eleven observers participated in this experiment. All of them had participated in Experiment 2, but they were still naive as to the purpose of the experiment.

### Stimuli

We used the video clips of simulated hot air flow as stimuli. Here, we manipulated distortion factors in 20 levels (from 10 to 200 in steps of 10). The distortion factor manipulation drastically affected the magnitude of image deformation; the larger distortion factor was, the larger the deformation became. As stimuli in the control condition, we also used the video clips of water flow. In addition to the ‘Rocks’ described above, we used ‘Grass’ images (the rightmost image in Supplementary Figure 1) as background in both the water and hot air simulations.

### Procedure

As in Experiments 2 and 3, we asked the observers to sequentially rate their impressions of both the water and hot air. Each observer received 168 trials consisting of 2 backgrounds × 20 levels of distortion factors × 4 repetitions of simulated hot air, and an additional 2 backgrounds × 4 repetitions of simulated water. We collapsed the data across background and repetitions in the analysis.

## Additional Information

**How to cite this article:** Kawabe, T. and Kogovšek, R. Image deformation as a cue to material category judgment. *Sci. Rep.* **7**, 44274; doi: 10.1038/srep44274 (2017).

**Publisher's note:** Springer Nature remains neutral with regard to jurisdictional claims in published maps and institutional affiliations.

## Supplementary Material

Supplementary Information

Supplementary Video 1

Supplementary Video 2

Supplementary Video 3

Supplementary Video 4
