# Supplementary material for: Image deformation as a cue to material category judgment
Source: Sci Rep. 2017 Mar 9;7:44274. doi: 10.1038/srep44274 (PMC5343573; doi:10.1038/srep44274)
Supplement: Supplementary Information [file srep44274-s1.pdf]

**Supplementary information for**
**Image deformation as a cue to material category judgment**

Takahiro Kawabe[1,§,*] & Rok Kogovšek[1, 2, §]

[1]NTT Communication Science Laboratories, Nippon Telegraph and Telephone Corporation, Japan

[2]Faculty of Computer and Information Science, University of Ljubljana, Slovenia

[§]These authors equally contributed to this work.

Submitted to *Scientific Reports*

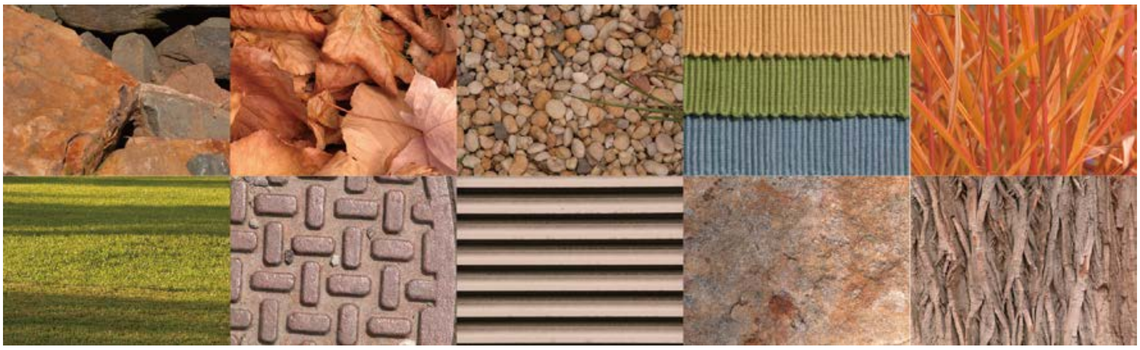

**Supplementary Figure 1.** Background images as used in the simulation of hot air and water flows. The images were taken from the McGill Calibrated Colour Image Database http://tabby.vision.mcgill.ca/.

**Video legends**

**Video 1**. Sample clips of stimuli as used in Experiment 2. Background images were taken from the McGill Calibrated Colour Image Database http://tabby.vision.mcgill.ca/.

**Video 2**. Sample clips of stimuli as used in Experiment 3. Background images were taken from the McGill Calibrated Colour Image Database http://tabby.vision.mcgill.ca/.

**Video 3**. Sample clips of stimuli as used in Experiment 4. Background images were taken from the McGill Calibrated Colour Image Database http://tabby.vision.mcgill.ca/.

**Video 4**. The role of image blurriness in the simulation of hot air. Background images were taken from the McGill Calibrated Colour Image Database http://tabby.vision.mcgill.ca/.
